# Supplementary material for: CD38 Expression by Antigen-Specific CD4 T Cells Is Significantly Restored 5 Months After Treatment Initiation Independently of Sputum Bacterial Load at the Time of Tuberculosis Diagnosis
Source: Front Med (Lausanne). 2022 Apr 15;9:821776. doi: 10.3389/fmed.2022.821776 (PMC9051241; doi:10.3389/fmed.2022.821776)
Supplement: Supplementary file 1 [file Data_Sheet_1.docx]

**Supplementary table 1**

| **Primer** | **Covering sequence** |
| --- | --- |
| 16S-Fw | 5'-GTGATCTGCCCTGCACTTC-3' |
| 16S-Rv | 5'-ATCCCACACCGCTAAAGCG-3' |
| IC-Fw | 5'-GCACAGGGTTGATGTTGGTATTGTC-3' |
| IC-Rv | 5'-CAAATGAGAAATAGCCCTCACTGCAAG-3' |
| **Probes** | **Reporters, covering sequences and quencher** |
| 16S-FAM | 5'6FAM-AGGACCACGGGATGCATGTCTTGT-3'BHQ1 |
| IC-JOE | 5'-JOE-GCAGGGTCCTCAGTTCTAGCAGGCTCCA-3'BHQ1 |

**Supplementary figure 1**

**
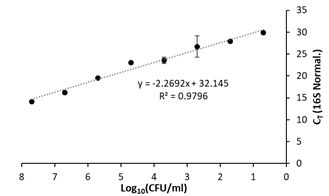
A B**

**
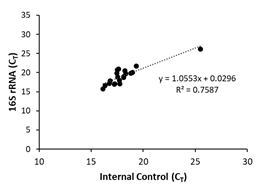
**

1. Linear regression analysis between cycle threshold (Ct) values of 16S rRNA of an H37Rv culture and the spiked internal control across 24 extraction/amplification replicates resulted in a slope of 1.0553. B) Linear regression analysis between IC-normalized 16S rRNA Ct values and bacterial concentration for an H37Rv dilution series (ranging from 5×10^7^ to 5 CFU/ml) processed in triplicate. The computed equation displayed on the plot was used to interpolate the bacterial load within the investigated sputum specimens from the normalized MBLA Ct values. CFU, colony-forming units.

**Supplementary figure 2**

Box plot representation of A-C) CD27 or D-F) CD38 biomarker expression by *Mtb*-specific CD4 T cells (CD27- or CD38-based TAM-TB) and A, D) time to culture positivity in weeks, or B, E) solid culture intensity grades from the same sputum specimen and C, F) patient’s clinical TB score at time of diagnosis (p values from one way ANOVA test for trend).
